# Mapping Enzyme Activity in Living Systems by Real-Time Mid-Infrared Photothermal Imaging of Nitrile Chameleons

**DOI:** 10.21203/rs.3.rs-2592139/v1

**Published:** 2023-03-01

**Authors:** Hongjian He, Jiaze Yin, Mingsheng Li, Xinyan Teng, Meng Zhang, Yueming Li, Zhiyi Du, Bing Xu, Ji-Xin Cheng

**Affiliations:** 1 Department of Electrical and Computer Engineering, Boston University, Boston, MA 02215, USA.; 2 Photonics Center, Boston University, Boston, MA 02215, USA.; 3 Department of Biomedical Engineering, Boston University, Boston, MA 02215, USA.; 4 Department of Chemistry, Boston University, Boston, MA 02215, USA.; 5 Department of Mechanical Engineering, Boston University, Boston, MA 02215, USA.; 6 Department of Chemistry, Brandeis University, Waltham, MA 02453, USA

## Abstract

Enzymes are vital components in a variety of physiological and biochemical processes. Participation of various enzyme species are required for many biological events and signaling networks. Thus, spatially mapping the activity of multiple enzymes in a living system is significant for elucidating enzymatic functions in health and connections to diseases. Here, we report the development of nitrile (C≡N)-tagged enzyme activity reporters, named nitrile chameleons for the shifted peak between substrate and product. By real-time mid-infrared photothermal imaging of the enzymatic substrates and products at 300 nm resolution, our approach can map the activity distribution of different enzymes and quantitate the relative catalytic efficiency in living cancer cells, C. elegans, and brain tissues. An important finding is the direct visualization of caspase-phosphatase cooperation during apoptosis. Our method is generally applicable to a broad category of enzymes and will advance the discovery of potential targets for diagnosis and drug development.

Enzymes, as efficient and specific catalysts of a variety of biochemical reactions, play critical roles in literally all biological processes. Abnormal enzyme activity is found in many diseases, including cancer^1, 2^ and neurodegeneration^3, 4^. Precise spatial and temporal regulation of enzyme activity is crucial to the successful operation of cellular machineries.^5^ Thus, it is significant to spatially map and quantify the activity of enzymes, preferentially in living organisms, for understanding the functions and roles of enzymes in health and disease. Notably, many biological events and signaling pathways are accomplished by the cooperation of multiple enzyme species. A typical example is programmed cell death, in which a collection of enzymes, such as caspase-3, 7, 8 and 9, are involved to execute apoptosis in one cell. Clearly, the attempts to map the activity of singular enzyme in one area of intertest (AOI) is insufficient to understand the cooperation among different enzymes in a complicated biological system. This gap urges the development of approaches that are capable of spatially profiling the activity of different enzymes in the same AOI at single cell level and in vivo for elucidating the links of multiple enzymes to life and pathophysiological processes.

Traditionally, visualizing enzyme activity is performed by synthetic fluorogenic substrates.^6−8^ The emission intensity of fluorogenic probes positively associates with enzyme activity. However, current fluorogenic probes fail to provide spatial information because the water-soluble fluorophores tend to diffuse away from the reaction sites.^9^ Recently, self-immobilizing fluorogenic reporters have been developed to locate enzyme activities in dynamic environments.^10^ In principle, the enzymatic reactions generate highly reactive fluorescent modalities to react with the nucleophiles in the targeted enzyme, which enables the retention of fluorophores on enzyme. However, the covalent connection of bulky fluorophores to enzymes increases the risk of perturbing the function of enzymes^11, 12^ and consequently disrupting the downstream signaling and cellular activity. Thus, the potential enzyme-silencing effects of self-immobilizing strategy diminish its application for mapping the activities of multiple enzymes in living systems.

A further development of fluorescent probes to profile enzyme activity in living organism derives from in-situ enzymatic noncovalent synthesis (ENS)^13^ of small molecules into non-diffusive nanostructures to allow the retention of fluorescent modalities at reaction sites.^14, 15^ Notably, fluorophores are critical to a successful mapping of enzyme activity via ENS-based fluorescent probes. Fluorophores with bulky structure, strong hydrophobicity, or charges (positive or negative) directly influence the dynamics, biodistribution, self-assembly and cellular uptake of the probes, even cause artifacts such as nonspecific binding.^16−18^ These filter criteria largely reduce the scope of fluorophore candidates for the ENS-based fluorescent probes. Given the broad-band emission spectrum, therefore, current research using such strategy mostly focuses on the mapping of singular enzyme^19^, which is barely enough to elucidate the cooperation between different enzymes in a biological process.

Beyond fluorescence, other technologies have achieved great advances in mapping enzyme activity.^20^ Enzyme-activated magnetic resonance sensors have been developed to image enzyme activity in cells and in vivo using magnetic resonance imaging (MRI).^21^ Although this method succeeds in locating enzyme activity in tissue, it hardly provides spatial details at subcellular level due to poor spatial resolution. Instead of imaging synthetic probes, mass spectrometry imaging (MSI) has been explored for assaying the spatial distribution of enzyme activity in tissue by direct detection of endogenous metabolites.^22^ Despite high sensitivity and spatial resolution, this approach is not applicable to living cells. Collectively, while tremendous efforts have been made towards the localization and quantification of enzyme activity, none realizes high-resolution mapping of the activity of multiple enzymes in living subjects.

Addressing this challenge requires innovations not only in imaging system but also in chemistry. Here, we present a method for visualizing enzyme activities via real-time mid-infrared photothermal (MIP) imaging of a category of nitrile-tagged enzyme activity probes. The recently developed MIP microscope enables infrared absorption chemical imaging with submicron spatial resolution by sensing the photothermal effect induced by mid-infrared absorption with a visible light.^23, 24^ Importantly, such indirect measurement mitigates the huge water absorption issue and allows mid-infrared imaging in aqueous environment with high contrast.^25^ The reported sample-scan MIP microscope needs a pixel dwell time ranging from 500 μs to 10 ms,^23, 24^ making it difficult to resolve the dynamics of living systems. To improve the MIP imaging speed, widefield approach using camera as the sensor has been developed for spatially multiplexed detection.^26^ However, this method works with low sensitivity to resolve signal over the shot noise due to the limited photon capacity of camera pixels.^26^ To address these difficulties, we have developed a laser-scan MIP microscope with high speed and high sensitivity. In this new system, a synchronized laser scanning scheme is exploited for high-speed imaging. Both visible probe and IR beam are turned by galvo mirrors for fast scanning, enabling a pixel dwell time as short as a few μs. We further optimize the IR excitation parameter to minimize water background in imaging aqueous specimens and achieve excellent MIP contrast with IR pulse duration around 50 ns. These efforts collectively allow real-time MIP imaging of living systems.

While label-free MIP microscopy succeeds in visualizing the abundant cellular components (e. g. proteins and lipid droplets),^23, 24^ direct observation of a specific enzymatic reaction remains difficult because the native MIP signals from products are likely immersed into the strong background generated by other biomolecules. To address this challenge, we synthesized a kind of nitrile-tagged bioorthogonal enzyme activity probes. The vibration frequency of the nitrile group (C≡N) differs from those of endogenous functional groups, thus exhibiting unique IR absorbance in the cell-silent region (1800~2800 cm^−1^).^27^ Moreover, the IR absorbance of C≡N is narrowband and spectrally tunable through chemical and physical approaches, enabling multiplexed detection.^28^ Thus, adopting C≡N as biochemical reaction reporters is promising for bio-orthogonal detection of multiple enzyme activities in living systems. Here, we synthesized a serial of nitrile-tagged enzyme activity probes, named as nitrile chameleons. The C≡N probes generate reaction-activatable MIP spectral shifts which signify enzymatic reactions. These probes also perform in-situ ENS^13^ for mapping enzyme activity.

Through MIP imaging of nitrile chameleons, we observed the biodistributions of phosphatase and caspase-3/7 activity in living cancer cells at sub-micrometer resolution. We further evaluated the effect of inhibitors on enzyme catalytic efficiency. The synchronous mapping of the activity of different enzymes suggests a positive coordination between phosphatase and caspase-3/7 in apoptosis. For in vivo study, we profiled the activity of phosphatase and caspase, individually and simultaneously, in living C. elegans and mouse cerebral cortex.

## Results

### Performance of a laser-scanning MIP microscope

**Figure 1.**
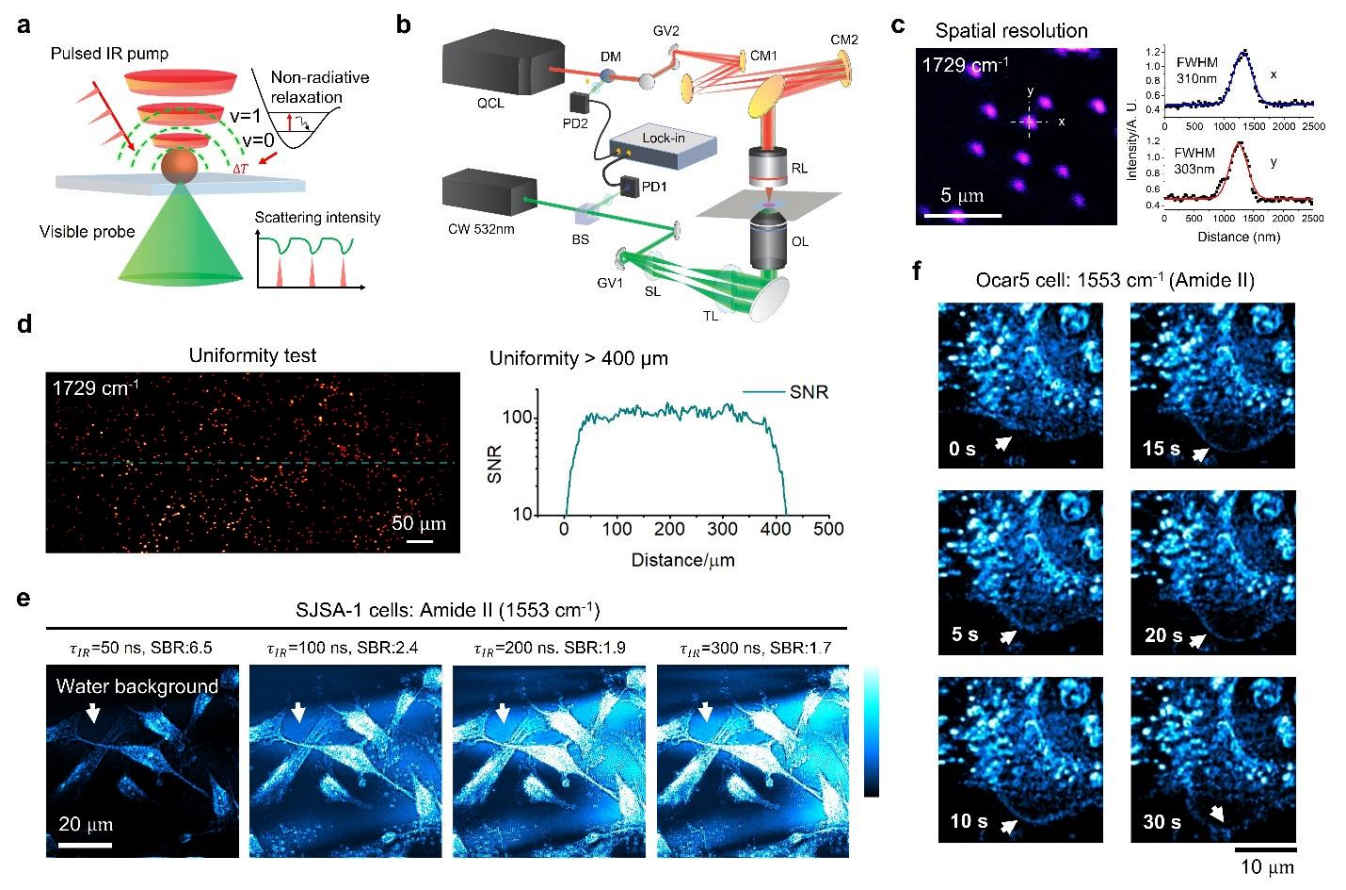
A laser-scan mid-infrared photothermal (MIP) microscope for real-time bond-selective imaging of living cells at 300-nm spatial resolution. (a) Principle of MIP detection. (b) Schematic illustration of imaging setup. (c) Spatial resolution characterized with 200-nm diameter PMMA particles. (d) Uniformity characterization by MIP imaging of 500-nm diameter PMMA particles SNR. (e) MIP imaging of proteins in cancer cells with different IR excitation pulse width. (f) Live cell MIP imaging of protein dynamics (1553 cm^−1^, amide II) in cancer cells.

Figure 1a illustrates the principle of mid-infrared photothermal imaging. A mid-infrared light excites the chemical bond through vibrational transition. The vibrational energy is deposited into the sample which subsequently experiences a local temperature rise. The temperature escalation introduces several photothermal effects such as thermal expansion and refractive index alteration. Those changes influence the scattering of a visible probe, which is adopted as the photothermal signal. Previously, MIP microscopes relied on sample scan with a pixel dwell time of 500 μs or longer, which is insufficient to capture dynamics in a living system. To increase the MIP imaging speed, we designed and built a laser-scan MIP microscope (Figure 1b) that provides a much faster pixel resolving speed one the order of a few microseconds (see methods). For providing high spatial resolution, counter-propagating geometry is applied. Specifically, visible beam is focused via a water immersion objective lens with 1.2 NA, while the infrared (IR) beam is focused by a reflective objective with 0.5 NA. The spatial resolution is characterized by imaging of PMMA particles with 200 nm diameter (Figure 1c). Calculated resolution reached 300 nm, approaching to the theoretical diffraction limit of 221 nm. Because MIP is a pump-probe technique, IR and visible beams are synchronously scanned by two pairs of galvo mirrors providing a uniform field of view over 400 μm (Figure 1d). For live cell imaging, we optimized the IR excitation pulse width for maximizing the signal to water background ratio. The photothermal dynamics of water background is known to have a large decay constant *τ*_*B*_ due to the large heat capacity. The decay constant *τ*_*S*_ for the signal from organelles is usually smaller. We harness such difference to enhance the signal to background contrast by changing the heating pulse width *τ* (see methods). Experimentally, we compared the influence of heating pulse duration on the MIP contrast of a live cell. Results show that a pulse width of 50 ns provides the highest SBR with negligible photodamage (Figure 1e and Supplementary Figure 1). Thus, we demonstrated live cell MIP imaging of protein dynamics (Figure 1f and Supplementary Video 1) and kept the conditions for the following experiments untiled specifically noted.

### Nitrile-tagged enzymatic activity probes

**Figure 2.**
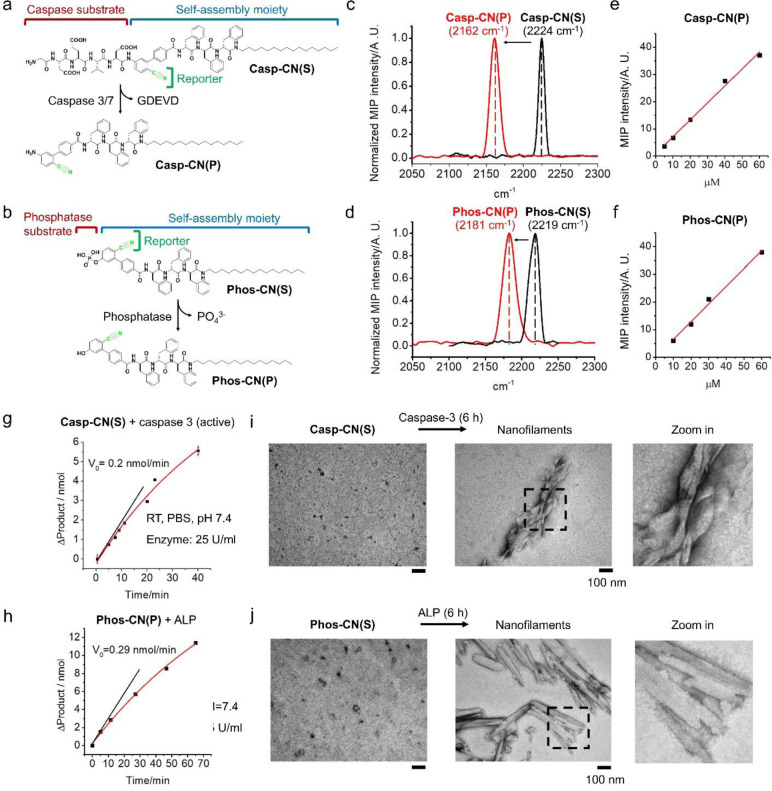
Development of nitrile chameleons for mapping specific enzyme activity. (a) Molecular structures of **Casp-CN(S)** and the enzymatic product **Casp-CN(P)** (b) Molecular structures of **Phos-CN(S)** and the enzymatic product **Phos-CN(P)** (c) MIP spectra of **Casp-CN(S)** and the enzymatic product **Casp-CN(P)**, 50 mM in DMSO (d) MIP spectra of **Phos-CN(S)** and the enzymatic product **Phos-CN(P)**, 50 mM in DMSO (e) and (f) MIP signal intensity of (e) **Casp-CN(P)** and (f) **Phos-CN(P)** at different concentration concentrations in DMSO. (g) Time-dependent formation of **Casp-CN(P)** catalyzed by active caspase 3 (25 U/mL) (h) Time-dependent formation of **Phos-CN(P)** catalyzed by alkaline phosphatase (ALP, 0.5 U/mL) (i) TEM images of the nano-assemblies formed by **Casp-CN(S)** before and after the addition of active caspase 3 (25 U/mL, 6 h) (j) TEM images of the nano-assemblies formed by **Phos-CN(S)** before and after the addition of alkaline phosphatase (ALP, 0.5 U/mL 6 h)

We constructed two probes that selectively map the activity of phosphatase and caspase in living subjects, because both phosphatase and caspase are critical to the survival and death of cells. The enzyme activity probes comprise of an enzymatic substrate, a nitrile group (C≡N) as reaction reporter, and a self-assembly moiety (Figure 2a and 2b). The nitrile-tagged probes for mapping the activity of caspase and phosphatases are named **Casp-CN(S)** and **Phos-CN(S)**, respectively. The enzymatic products are named **Casp-CN(P)** and **Phos-CN(P)**, respectively. Specifically, we conjugated the substrate of caspase-3/7 (DEVD) or the general substrate of phosphatase (phosphate ester) to the para position of benzonitrile. The para-position atoms (O or N) exhibit electron-donating effect while C≡N withdraws the electrons from the phenyl group. The enzyme-catalyzed cleavage of substrates alters the electronic donation from the para-position atoms, thus, significantly changing the electron density as well as the bond vibration of C≡N. Consequently, in MIP spectra, the C≡N of enzymatic products are spectrally separated from those before enzymatic reactions. The MIP signal intensity of the C≡N of enzymatic products positively correlates with the level of enzyme activity. Importantly, this reaction-activatable peak shift of C≡N not only reports the occurrence of enzymatic reactions, but also procures a bioorthogonal detection of enzymatic products via MIP imaging in the cell-silent region. For mapping the biodistribution of enzyme activity, we further conjugated a self-assembly moiety to the probes (Figure 2a and 2b) to allow in situ ENS for conserving the products at the reaction sites. The ENS of probes also amplifies imaging contrast via aggregation-enhanced responsiveness^29^, a phenomenon likely caused by concentrating the targeted molecules within the volume of imaging. We named this category of probes as nitrile chameleons because of the “spectral shift” of C≡N.

As shown in Figure 2c, the MIP spectra of the C≡N bond of **Casp-CN(S)** and **Casp-CN(P)** (50 mM, in DMSO) exhibit a sharp peak at 2224 and 2162 cm^−1^, respectively, while **Phos-CN(S)** and **Phos-CN(P)** (50 mM, in DMSO) show narrow-band MIP spectrum of C≡N with a peak at 2219 and 2181 cm^−1^, respectively (Figure 2d). These distinctive peaks of C≡N guarantee multi-color MIP imaging of the nitrile probes and the corresponding enzymatic products. Moreover, the MIP signal intensity of the C≡N bond in enzymatic products linearly proportionates to the concentrations with a limit of detection (LOD) around 5 μM (Figure 2e, 2f and Supplementary Figure S2). Notably, this sensitivity is higher than SRS imaging of the C≡C bond in EdU^30^ by three orders of magnitude, thus providing highly sensitive detection of enzyme activities. We evaluated the enzymatic conversion efficiency of **Casp-CN(S)** by caspase-3 (active) and **Phos-CN(S)** by alkaline phosphatase (ALP) via monitoring the production formation overtime (Figure 2g and 2h). Time-course analysis of product formation reveals that the initial speed (v_0_) is 0.2 nmol^−1^min^−1^ for **Casp-CN(S)** by caspase-3, and 0.29 nmol^−1^min^−1^ for **Phos-CN(S)** by ALP. The v_0_ reduces tolerantly compared with that performed by standard substrates (Ac-DEVD-pNA, 0.33 nmol/min, and P-nitrophenyl phosphate, 0.53 nmol/min) in identical conditions (Supplementary Figure 3), probably due to a less electron-withdrawing effect of C≡N versus the nitro substitution (NO_2_) in standard substrates.^31^ Transmission electron microscopy (TEM) images of **Casp-CN(S)** and **Phos-CN(S)** (50 μM, in PBS) exhibit sparsely distributed small nanoparticles formed by the self-assembly of the probes (Figure 2i and 2j). Bulky nanofilaments are observed in TEM images after 6-hour incubation of the probes with corresponding enzymes (Figure 2i and 2j). This result verifies the ENS of nitrile chameleons which permits spatial mapping of enzyme activities.

### Real-time MIP imaging of nitrile chameleons to map enzyme activity in living cells

**Figure 3.**
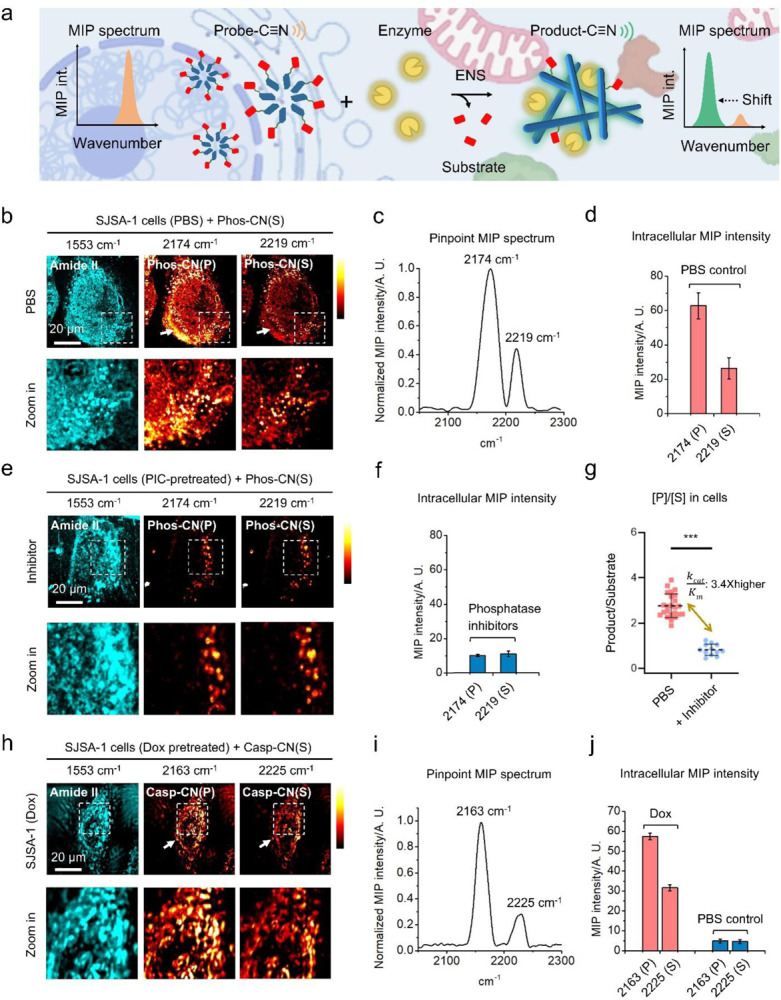
Real-time MIP imaging of nitrile chameleons generates the activity maps of caspase 3/7 and phosphatase in living cells. (a) Schematic illustration of the principle of enzyme activity mapping by real-time MIP imaging of nitrile chameleons (b) MIP images of phosphatase activity profile in living SJSA-1 cells (c) Pinpointed MIP spectrum (indicated by arrow) (d) Quantification of MIP signal intensity of **Phos-CN(S)** and **Phos-CN(P)** in cell (e) MIP images of phosphatase activity profile in phosphatase inhibitor-pretreated SJSA-1 cells (f) Quantification of MIP signal intensity of **Phos-CN(S)** and **Phos-CN(P)** in phosphatase inhibitor-pretreated SJSA-1 cells (g) Statistic of product-to-substrate ratio ([P]/[S]) in the cells from PIC-pretreated and PIC-free groups. (h) MIP images of caspase 3/7 activity profile in Doxorubicin-pretreated SJSA-1 cells (i) Pinpoint MIP spectrum (indicated by arrow) (j) Quantification of MIP intensity of **Casp-CN(S)** and **Casp-CN(P)** in Dox-pretreated cells and the cells from Dox-free control group

The principle is illustrated in Figure 3a. The probes enter cells and react with corresponding enzymes, generating products that exhibit MIP signal at new wavenumbers. The MIP signal intensity from products is positively relevant to enzyme activity. Meanwhile, the ENS of probes produces non-diffusive nanofilaments which not only uncover the reaction sites, but also enhance the imaging contrast by aggregation-enhanced responsiveness^29^. We firstly used this approach to map the activity of phosphatase in SJSA-1 cells, an osteosarcoma cell line with high phosphatase expression, especially ALP. After the treatment of **Phos-CN(S)** (50 μM, 4 h), live-cell MIP imaging at 1553 cm^−1^ (amide II) reveals the profiles of protein and peptide in SJSA-1 cells (Figure 3b). A pinpointed (indicated by arrow) MIP spectrum inside the cell exhibits a sharp peak at 2174 cm^−1^ arising from the C≡N of the dephosphorylated products (**Phos-CN(P)**), and a smaller peak at 2219 cm^−1^ originating from the C≡N of **Phos-CN(S)** (Figure 3c). The wavenumbers of C≡N drift slightly in cells compared to in DMSO (Figure 1d) probably due to solvent effect^32, 33^. MIP images of living SJSA-1 cells at 2174 cm^−1^ show strong signal from **Phos-CN(P)**, while the intracellular MIP signal from **Phos-CN(S)** at 2219 cm^−1^ is weaker (Figure 3b and 3d). These results suggest that the phosphatases in SJSA-1 efficiently convert **Phos-CN(S)** to **Phos-CN(P)**, disclosing a high intracellular phosphatase activity. Instead of a diffusive display, **Phos-CN(P)** exposes sophisticated subcellular structures varied by MIP intensity in the mapping (Figure 3b), indicating a heterogeneous biodistribution of phosphatase activity. Colocalization between **Phos-CN(S)** and **Phos-CN(P)** is observed (Figure 3b), suggesting the coassembly between probes and products into non-diffusive nanofilaments during ENS.^34−36^ However, the dissolved portion of **Phos-CN(S)** is hardly visible due to the lack of aggregation-enhanced responsiveness. Integral MIP-fluorescence imaging validates a good colocalization (Pearson’s r=0.7, Supplementary Figure 4) between the MIP signal from **Phos-CN(P)** and the immunofluorescence from phosphatase antibodies (e.g., Anti-ALP), confirming the high spatial accuracy of mapping. As controls, we hardly observed MIP contrast in the same cells at off-resonance (2050 cm^−1^), neither in the cells without **Phos-CN(S)** treatment at 2174 cm^−1^ (Supplementary Figure 5). These results collectively confirm a successful mapping of phosphatase activity in living SJSA-1 cells by real-time MIP imaging of nitrile chameleon. The phosphatase inhibitor cocktails (PIC)-pretreated SJSA-1 cells incubated with **Phos-CN(S)** show a punctate distribution of **Phos-CN(P)** and **Phos-CN(S)** with much weaker MIP signals (Figure 3e and 3f) compared to the cells from inhibitor-free group (Figure 3b and 3d), which agrees with the phosphatase activity inhibition. These results validate that **Phos-CN(S)** can spatiotemporally unveil phosphatase activity change in cells.

Since PIC treatment hardly changes the phosphatase expression level (e. g. ALP) in cells (Supplementary Figure 6), according to Michaelis-Menten equation (see methods), the PIC-induced degree of inhibition on the average enzyme catalytic efficiency (k_cat_/K_M_) of phosphatase in cells can be approximately quantified through comparing the product-to-substrate ratio ([P]/[S]), determined by the ratio of intracellular MIP intensity at 2174 cm^−1^ versus 2219 cm^−1^, between the cell populations from PIC-pretreated and inhibitor-free groups. Statistically, the average [P]/[S] in PIC-pretreated cells is 3.4 times lower than the one in PIC-free group (Figure 3g), indicating that the average k_cat_/K_M_ of the phosphatase in SJSA-1 cells decreases 70% through PIC treatment.

Other than phosphatase, we profiled caspase-3/7 activity in living cancer cells. Doxorubicin (Dox), a commonly used chemotherapeutic drug, efficiently induces apoptosis in SJSA-1 cells for activating caspase-3/7 (Supplementary Figure 7). Like the mapping of phosphatase activity, real-time MIP imaging at 1553 cm^−1^ generates the map of proteins and peptides in the apoptotic cells (Figure 3h). After the incubation with **Casp-CN(S)** (50 μM, 6 h), a pinpointed (indicated by arrow) MIP spectrum in the cells exhibits a sharp peak at 2163 cm^−1^ arising from the C≡N in **Casp-CN(P)** and a lower peak originating from the C≡N in **Casp-CN(S)** at 2225 cm^−1^ (Figure 3i), validating the chemical compositions. In comparison, MIP images of the Dox-pretreated SJSA-1 cells show intensive MIP signal from **Casp-CN(P)** at 2163 cm^−1^ and less signal from **Casp-CN(S)** at 2225 cm^−1^ with detailed spatial information (Figure 3h). These results indicate a high caspase-3/7 activity in the Dox-pretreated SJSA-1 cells. The colocalization between **Casp-CN(S)** and **Casp-CN(P)** (Figure 3h) is probably caused by the coassembly into nanofilaments during ENS.^34, 35^ Tandem MIP-fluorescence imaging validates a high spatial correlation between the MIP signal from **Casp-CN(P)** and the immunofluorescence from Caspase-3 (active) antibody (Pearson’s r=0.72, Supplementary Figure 3). These results confirm a precise mapping of caspase-3/7 activity in apoptotic cells. While the nitrile chameleons are noncytotoxic (Supplementary Figure 8), incubating the Dox-free cells with **Casp-CN(S)** show weak but non-zero MIP signal from **Casp-CN(P)** (Figure 3j and Supplementary Figure 9), indicating a nonapoptotic function of caspase-3/7 activity.^37−39^

### Multicolor MIP imaging of nitrile chameleons reveals caspase-phosphatase cooperation

**Figure 4.**
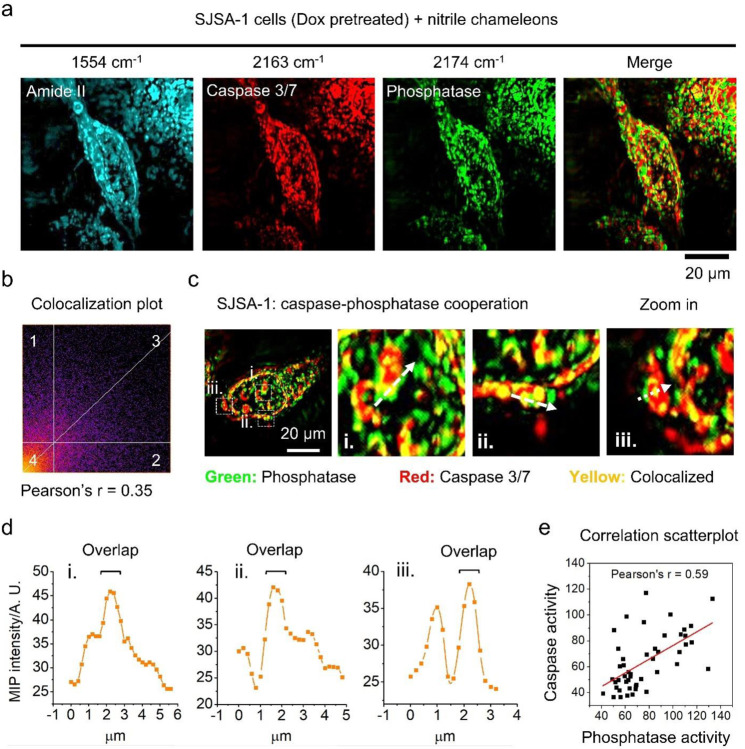
Multicolor MIP imaging of nitrile chameleons provides evidence of caspase-phosphatase cooperation in apoptosis. (a) Simultaneous visualization of phosphatase and caspase-3/7 activity profile in Dox-pretreated SJSA-1 cells. (b) Colocalization analysis of the mapping in (a). (c) Study of spatial interaction between phosphatase and caspase-3/7 in Dox-pretreated SJSA-1 cells. (d) Intensity plot of **Phos-CN(P)** along the arrows in (c). (e) Correlation scatterplot of caspase-3/7 and phosphatase activity in Dox-pretreated SJSA-1 cells

Phosphatases act key roles in modulating the activities of numerous proteins in cells, and caspases are essential enzymes for apoptosis. To explore phosphatase-caspase cooperation in programed cell death, we simultaneously disclosed the activity distribution of phosphatase and caspase-3/7 in apoptotic cancer cells by MIP imaging of **Phos-CN(P)** and **Casp-CN(P)**, because the yield of enzymatic products is positively associate with enzyme activity. The MIP spectra of C≡N in **Phos-CN(P)** and **Casp-CN(P)** in cells appear as narrow bands at unique wavenumbers (Figure 3c and 3i), enabling multispectral imaging of these two enzymatic products. As shown in Figure 4a, MIP imaging of proteins and peptides at 1553 cm^−1^ (amide II) displays the location and morphology of the Dox-pretreated SJSA-1 cells. After incubating the apoptotic cells with **Phos-CN(S)** and **Casp-CN(S)**, strong MIP signals from the C≡N of **Phos-CN(P)** and **Casp-CN(P)** with fine textures are observed in cells (Figure 4a), suggesting a high-level activity of phosphatase and caspase-3/7 in the cells. The merge channel and colocalization analysis confirms a weak spatial correlation between the MIP signals from **Phos-CN(P)** and **Casp-CN(P)** (Pearson’s r=0.35, Figure 4b), validating that MIP imaging of nitrile chameleons not only visualizes, but also spatially and spectrally distinguishes the activity distribution of different enzymes. Interestingly, while the activity maps of phosphatase and caspase-3/7 are mostly independent, sporadic coexistences were observed in the cells (Figure 4c). The chemical components were validated by pinpointed MIP spectrum (Supplementary Figure 10). This result indicates potential caspase-phosphatase interactions during apoptosis. It is reported that some members of phosphatase family, such as PTEN^40^ and PTP-PEST^41^, are potential substrates of caspase-3. Importantly, the caspase-3-catalyzed cleavage of PTP-PEST increases the catalytic activity of PTP-PEST and alters its scaffolding properties^41^. Notably, in the activity map of phosphatase and caspase-3/7, area with colocalization generally exhibit stronger MIP signal from **Phos-CN(P)** than the ambient, as revealed by the intensity plots alone the arrows (Figure 4d), indicating a higher phosphatase activity in the sites that are coupled with caspase 3/7 activity. Statistically, the activity level of phosphatase in SJSA-1 cell population positively associate with that of caspase-3/7 (Figure 4e). Thus, simultaneous mapping of phosphatase and caspase-3/7 activity probably localize the caspase-mediated regulation of the activity of phosphatase, likely PTP-PEST, in the apoptotic SJSA-1 cells. In addition to SJSA-1, we observed similar scenario in MIA PaCa-2 (Supplementary Figure 11), a cell line derived from pancreatic tumor. These results strongly support the notion that phosphatase plays an important role in regulation of programed cell death^42−45^, and thus suggest phosphatase a promising therapeutic target for cancer treatment.

### Multicolor in vivo MIP imaging of caspase and phosphatase activities.

**Figure 5.**
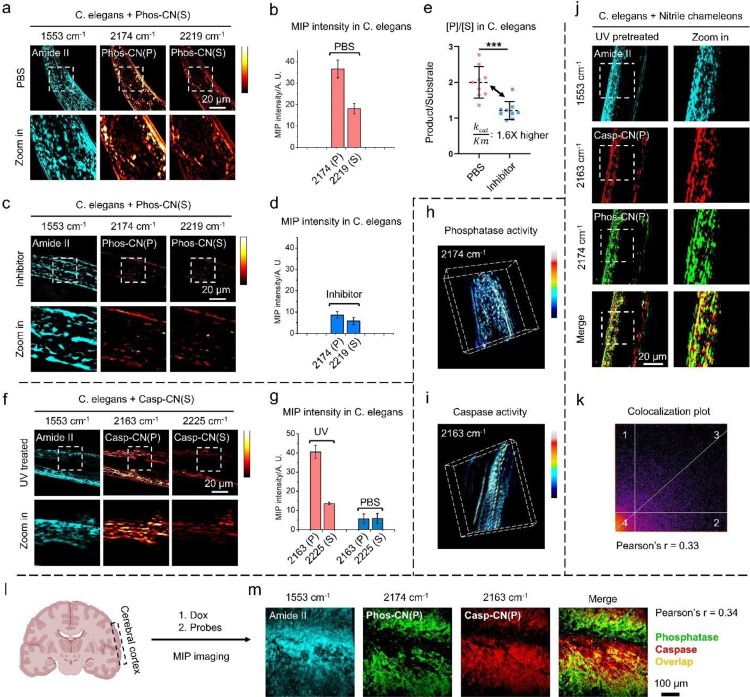
Multicolor in vivo MIP imaging of nitrile chameleons generates activity maps of caspase and phosphatase inside C. elegans. (a) MIP imaging of protein and phosphatase profile in living C. elegans (b) Quantification of the MIP signal intensity from **Phos-CN-(P)** and **Phos-CN-(S)** in the inhibitor-free C. elegans. (c) MIP imaging of protein and phosphatase activity profile in the PIC-pretreated C. elegans. (d) Quantification of the MIP signal intensity from **Phos-CN-(P)** and **Phos-CN-(S)** in the PIC-pretreated C. elegans (e) Statistic of phosphatase [P]/[S] values in the inhibitor-free C. elegans and PIC-pretreated C. elegans (f) MIP imaging of protein and caspase activity profile in UV-pretreated (0.5 mg/mL) C. elegans (g) Quantification of the MIP signal intensity from **Casp-CN-(P)** and **Casp-CN-(S)** in the UV-pretreated and drug-free C. elegans. (h) and (i) 3D reconstruction of (h) phosphatase activity and (i) caspase activity in C. e elegans. (j) Simultaneous activity mapping of phosphatase and caspase in UV-pretreated (0.5 mg/mL) C. elegans (k) Colocalization analysis of the mapping in (j) (l) and (m) Simultaneous mapping of phosphatase and caspase activities in Dox-pretreated (1 μM) mousee cerebral cortex sections.

Finally, we explored in vivo enzyme activity mapping in C. elegans and brain tissues via MIP imaging of the nitrile chameleons (Figure 5). After incubating **Phos-CN(S)** with C. elegans, real-time MIP imaging at 1553 and 2174 cm^−1^ reveal the biodistribution of proteins and **Phos-CN(P)** which expose phosphatase activity profile in C. elegans (Figure 5a). While C. elegans lacks ALP, the intensive MIP signal from the C≡N of **Phos-CN(P)** at 2174 cm^−1^ and a weaker one from **Phos-CN(S)** at 2219 cm^−1^ confirm an efficient enzymatic conversion (Figure 5a and 5b), suggesting a high activity from other phosphatase isoenzymes, such as PTP, in C. elegans.^46^ As controls, MIP contrast is hardly observed by off-resonance excitation, or in the C. elegans without nitrile chameleons treatment (Supplementary Figure 12). PIC significantly reduces the phosphatase activity in C. elegans, as revealed by the weaker MIP signal from **Phos-CN(P)** and **Phos-CN(S)** (Figure 5c and 5d). The average [P]/[S] obtained from the PIC-treated C. elegans versus the one from inhibitor-free group suggests a 38% decrease in the catalytic efficiency (k_cat_/K_M_) of the phosphatase (Figure e) in C. elegans via PIC treatment. Although the caspase (e. g. CED-3) of C. elegans may differ from the one of human, DEVD works as the substrate.^47^ We exposed C. elegans to UV radiation to induce apoptosis in C. elegans for caspase activation.^48^ After incubating the UV-irradiated C. elegans with **Casp-CN(S)**, real-time MIP imaging at 2163 cm^−1^ reveals the activity profile of the caspase in C. elegans (Figure 5f). The strong MIP signal from **Casp-CN(P)** and a lower signal from **Casp-CN(S)** at 2223 cm^−1^ confirm a high caspase activity in the UV-treated C. elegans (Figure 5f and 5g). Conversely, faint MIP signals from **Casp-CN(P)** and **Casp-CN(S)** were observed in the UV-free C. elegans (Supplementary Figure 13), indicating the existence of a weak caspase activity background in C. elegans. We further reconstructed the 3D activity profiles of phosphatase and caspase in the nematode (Figure 5h and 5i and Supplementary Video 2 and 3). Besides visualizing the enzyme activity individually, we concurrently mapped the activity of phosphatase and caspase in C. elegans through incubating the UV-irradiated C. elegans with nitrile chameleons followed by MIP imaging (Figure 5j). The merge channel and colocalization analysis show a mediocre overlapping between the MIP signals from **Phos-CN(P)** and **Casp-CN(P)** (Pearson’s r=0.33, Figure 5k), confirming the identification and differentiation of the activity profiles of diverse enzymes in C. elegans by MIP imaging of nitrile chameleons.

We further concurrently mapped the activity of phosphatase and caspase-3/7 in the sections of mice brains. Caspase-3/7 in fresh mice brains was activated through ex vivo incubation with Dox (1 μM, 24 h), simulating chemotherapy-induced neurotoxicity. After incubating the Dox-treated tissues with nitrile chameleons, MIP imaging produced clear activity maps of phosphatase and caspase-3/7 in the unfixed tissue sections of cerebral cortex (Figure 5l and 5m). The merged channel and colocalization analysis revealed a low spatial overlapping between **Phos-CN(P)** and **Casp-CN(P)** (Pearson’s r=0.34, Figure 5m). This supports the capability to identify and differentiate the activity distribution of various enzyme in cerebral cortex via MIP imaging of nitrile chameleons. As controls, little MIP contrast of C≡N is shown at off-resonance (2050 cm-1), neither in the tissues without the treatment of nitrile chameleons (Supplementary Figure 14).

## Discussion

An approach for mapping the activity of various enzymes in living systems is demonstrated. The mapping is achieved by bio-orthogonal chemical imaging of multiple reaction-activatable reporters on a high-speed mid-infrared photothermal imaging platform. Two innovations in instrumentation and chemistry, namely the development of a laser-scan MIP microscope and the synthesis of nitrile-tagged probes, made this method surpass traditional ways of enzyme activity imaging. Moreover, our approach endows the comparation of catalytic efficiency of specific enzymes between different cell populations, exceling conventional off-on probes^6, 49^ for imaging enzyme activity. An intriguing finding is the direct visualization of caspase-phosphatase cooperation during apoptosis inside cancer cells.

The laser-scanning MIP microscope developed in this work pushes the imaging speed to real-time. Together with the high sensitivity in bond-selective imaging, this technology allows the visualization of chemical dynamics inside a living system with submicron resolution. Such advantage promises broad biological applications. Additionally, the system is highly compatible with the fluorescence imaging modality by sharing the same visible scanning beam path for confocal detection. This feature permits multimodal imaging, including fluorescence-guided MIP and fluorescence-detected MIP^50^.

While we focus on caspase and phosphatase in this study, activity mapping of a broad category of enzyme species, such as esterase, kinase, and other proteases, can be done by developing more spectrally resolvable enzymatic reaction reporters. For example, introducing other functional groups with bio-orthogonal IR absorbance, such as azide (−N_3_)^51^ and alkyne (C≡C)^30^, or utilizing isotope-labeled triple bonds^28^ (e. g. C≡N^15^, C^13^≡N and C^13^≡N^15^) can substantially improve the imaging multiplexity. Additionally, enzyme activity profiling in other tissues, such as tumor, is worth exploration via delivering the nitrile-tagged probes through in situ administration, and bio-orthogonal MIP mapping of enzyme activities in tissue sections. Furthermore, the concept of nitrile chameleon, namely the reaction-activable spectral shift of C≡N, can be extended for detecting numerous metabolic activities, including pH, reactive oxygen species, membrane potential, and post-translational modification.

In summary, the method reported here promises a broadly applicable approach to map enzyme activity in living systems and acquire new perceptions on the functions of multiple enzymes in biological events, health regulation, and pathological developments. The holistic comprehension in the characters of multiple enzymes will greatly promote the discovery of potential drug targets for diagnostic and therapeutic applications.

## Methods

### Materials:

RPMI-1640 culture medium was purchased from Thermofisher Scientific. RPMI 1640 culture medium for SJSA-1 cell culturing was supplied by 10% (vol/vol) FBS and 1% (vol/vol) penicillin/streptomycin to the medium. DMEM culture medium was purchased from Thermofisher Scientific. DMEM culture medium for MIA PaCa-2 cell culturing was supplied by 10% (vol/vol) FBS and 1% (vol/vol) penicillin/streptomycin to the medium. Nematode Growth Medium (NGM) for preparing C. elegans suspension was purchased from Fisher Scientific. Phosphatase Inhibitor Cocktail II (PIC) was purchased from Sigma Aldrich. All amino acid derivatives involved in the synthesis were purchased from Fisher Scientific. N, N’-diisopropylethylamine (DIEA) and O-(1H-Benzotriazol-1-yl)-N,N,N’,N’-tetramethyluronium hexafluorophosphate (HBTU) were purchased from Fisher Scientific. Human recombinant active caspase 3 was purchased from Abcam (ab52101), and alkaline phosphatase was purchased from Sigma Aldrich (10713023001). All reagents and solvents were used as received without further purification unless otherwise stated.

### Laser-scan MIP microscope:

The laser-scan MIP microscope is built on an inverted microscope frame (IX73, Olympus). The visible probe is provided by a continuous-wave 532 nm (Sambda, HUBNER photonics). The mid-infrared pump is provided by a pulsed quantum cascade laser (MIRcat 2400, Daylight Solutions) with tunable range from 900 cm^−1^ to 2300 cm^−1^. Visible beam is scanned by a galvo mirror with 3 mm aperture (Saturn 1B, ScannerMax) and focused with a water immersion objective lens (1.2NA, 60X, Olympus). The IR beam is synchronously scanned with another pair of galvo mirrors (GVS001, Thorlabs) and focused on the same spot with a reflective objective (0.5NA, 40X, Thorlabs). For IR beam scanning, reflective conjugation with two concave mirrors is used for removing the chromatic aberration. Probe photonics are collected in both forward and backward directions. Their intensity is sensed by a silicon photodiode (DET100A, Thorlabs) connected with low-noise amplifier (SA230, NF cooperation). Amplifier signal is then sent to a lock-in amplifier (HF2LI, Zurich) for MIP signal demodulation.

### Signal versus background in a MIP microscope:

Such mechanism is described with temperature rising model where *S*_0_, *B*_0_ are the final amplitude that has been reached. The pulse width of IR excitation is *τ*. The thermal decay constant of signal and background are *τ*_*S*_, and *τ*_*B*_ accordingly. Based on the photothermal signal model proposed, the actual amplitude *S*, *B* from sample and background is then written as:

[1] $$S=S_{0}\left(1-e^{-\frac{\tau }{\tau_{S}}}\right)$$

[2] $$B=B_{0}\left(1-e^{-\frac{\tau }{\tau_{B}}}\right)$$

The signal to background ratio (SBR) is defined as:

[3] $$SBR=\frac{S_{0}}{B_{0}}\frac{1-e^{-\frac{\tau }{\tau_{S}}}}{1-e^{-\frac{\tau }{\tau_{B}}}}$$

SBR described in equation [3] is a function of heating pulse duration *τ* as the decay constant *τ*_*B*_, *τ*_*S*_ are different. SBR is scaled with $e^{\tau \left(\frac{1}{\tau_{B}}-\frac{1}{\tau_{S}}\right)}$. Since water is the major component of the culture medium, the decay constant *τ*_*B*_ is larger than *τ*_*S*_ due to the large water heat capacity. The decay constants for lipid droplet and water were measured to be 300 ns and 5.6 μs by mid-infrared photothermal dynamic imaging^52^. Thus, SBR decreases with the pulse duration. Moreover, a shorter pulse causes less photo damage compared to a longer pulse duration, making the setup more suitable for imaging living systems.

### Synthesis of nitrile probes:

The synthesis of self-assembly moiety was based on solid-phase peptide synthesis (SPPS). All probes were purified by Water Agilent 1100 HPLC system, equipped with an XTerra C18 RP column. Details of probe synthesis can be found in supplementary information.

### TEM imaging:

TEM images were taken on Morgagni 268 transmission electron microscope using negative staining. Samples were dropped on copper grids and dried by vacuum. Uranyl acetate (1 wt%, pH 4.5) was used to stain the samples for 3 times followed by washing using distilled water. Images were taken by lab members who were properly trained.

### Limit of Detection (LOD):

The LOD in this work is defined as the concentration that the MIP signal from the molecule equals to 3 times of the standard deviation of background noise. We obtained the MIP spectra (2050–2300 cm^−1^) of **Casp-CN(P)** and **Phos-CN(P)** dissolved in DMSO in different concentrations until the MIP signal intensity at the peaks from the molecules equals to 3 times of the standard deviation of background noise.

### Time-course monitoring of product formation:

The time-course formation of enzymatic products from standard substrates were done by following the manufacture’s protocol. Briefly, colorimetric substrates Ac-DEVD-pNA (100 μM) and pNPP (200 μM) were mixed with active caspase 3 (25 U/mL) and ALP (0.5 U/mL), respectively, at room temperature in PBS buffer. The UV absorbance at 405 nm was monitored over time by a UV-Vis spectrometer. Standard curves of enzymatic products were constructed to determine the product concentrations according to UV absorbance (Supplementary Figure S2).

The time-course formation of enzymatic products from nitrile chameleons were done by a similar way. Briefly, **Casp-CN(S)** (100 μM) and **Phos-CN(S)** (200 μM) were mixed with active caspase 3 (25 U/mL) and ALP (0.5 U/mL), respectively, at room temperature in PBS buffer. The UV absorbance of **Casp-CN(S)** (100 μM) mixed with caspase 3 was monitored at 380 nm over time by a UV-Vis spectrometer. The UV absorbance of **Phos-CN(S)** (200 μM) mixed with ALP was monitored at 340 nm over time by a UV-Vis spectrometer. Standard curves of enzymatic products were constructed to determine the product concentrations according to UV absorbance (Supplementary Figure S2).

### Sample preparation for MIP imaging:

For MIP imaging of live cells, SJSA-1 and MIA PaCa-2 cells were firstly seeded on CaF_2_ crystal substrates with a density of 1 × 10^5^/mL with 2 mL culture medium for overnight at 37 °C and 5% CO_2_. After cell attachment, cells were pretreated by conditions of interest. For examples PBS (control), Dox (2 μM, 24 h), and Phosphatase Inhibitor Cocktail II (0.25 μL in 1 mL medium, 24 h). After the treatment, cells were incubated with **Phos-CN(S)** (50 μM, 4 h) for phosphatase activity mapping, or **Casp-CN(S)** (50 μM, 6 h) for caspase activity mapping. Cells were then wash by PBS for 3 times, and sandwiched over a cover glass, followed by MIP imaging. For concurrent mapping the activity of two enzyme, solution of **Phos-CN(S)** (100 μM) and **Phos-CN(S)** (100 μM) were mixed at 1:1 ratio. Cells (2 μM Dox treated) were incubated with the mixture of probes (4–6 h), then wash by PBS for 3 times, and sandwiched over a cover glass, followed by MIP imaging. IR frequency: 1 MHz; IR power: 18 mW; Probe beam: 2 mW; IR pulse width: 50 ns; Pixel dual time: 10 μs.

For the optimization of laser pulse width, SJSA-1 cells were firstly seeded on CaF_2_ crystal substrates with a density of 1 × 10^5^/mL with 2 mL culture medium for overnight at 37 °C and 5% CO_2_. After cell attachment, cells were fixed by 4% formaldehyde to avoid shrinkage caused by heat damage while using long laser pulse width.

For MIP imaging of live C. elegans, wild-type C. elegans were grown at 20°C on NGM agar seeded with Escherichia coli OP50 by PBS washing. The C. elegans and e. coli suspension were centrifuged at 1000 rad/min for 30 s to separate C. elegans from e. coli. The C. elegans were resuspended in NGM followed by the treatment of conditions of interest. For examples, PBS (control), UV exposure (254 nm, until the movement of most C. elegans slow down) using a UV Stratalinker 2400, and PIC II treatment (1 μL PIC in 1 mL). After that, nitrile chameleons (50 μM) were mixed with C. elegans in NGM and incubated for 4–6 hours. The C. elegans were then washed by centrifuged in PBS for 3 time and sandwiched between CaF_2_ crystal substrate and cover glass for MIP imaging.

For 3D reconstruction of enzyme activity map in C. elegans, wild-type C. elegans were grown at 20°C on NGM agar seeded with Escherichia coli OP50 by PBS washing. The C. elegans and e. coli suspension were centrifuged at 1000 rad/min for 30 s to separate C. elegans from e. coli. The C. elegans were resuspended in NGM followed by the treatment of conditions of interest. The C elegans were then fixed by 4% formaldehyde to prevent movement during the 3D reconstruction.

For MIP imaging of cerebral cortex, the mice were anesthetized by isoflurane. Check the mice with a tail pinch, and quickly decapitate the mice. Open the scalp and skull with scissors, and carefully scoop out the brains. Place the brains in PBS at room temperature and wash the brains by PBS. Brain tissues were then cultured in 49 mL Hibernate-E Medium + 0.25 mL Gentamicin + 1 mL B-27 Plus Supplement + 50 uL Amphotericin B at 37 °C. Dox was then added into the medium to a final concentration of 1 μM. The brains were further incubated for 24 h. The brains were transferred to new culture medium containing Casp-CN(S) and Phos-CN(S) (50 μM) for 4 hours at 37 °C. After treatment, the tissue was sliced to coronal sections with a thickness of 100 μm using an Oscillating Tissue Slicer (OST-4500, Electron Microscopy Sciences). Brain slices were gently transferred by a brush and sandwiched between a CaF_2_ substrate and a cover glass for MIP imaging. The protocol of animal experiments was approved by BU IACUC (PROTO201800534).

### Enzyme catalytic efficiency:

k_cat_ is the first-order rate constant that determines the reaction rate when the enzyme is fully occupied at a saturating concentration of the substrate. K_M_ is Michaelis constant of the enzyme. It is the concentration of substrate which permits the enzyme to achieve half of the maximum speed. k_cat_/K_M_ measures the catalytic efficiency of a given enzyme. According to Michaelis-Menten kinetics rate, when [S]<<K_M_:

[4] $$\frac{d[P]}{dt}=v=\frac{\text{kcat}}{KM}[E][S]$$

It is reported that the K_M_ of Homo sapiens tissue-nonspecific alkaline phosphatase (wild type) and placental alkaline phosphatase is 210 and 800 μM (Numa, Natsuko, et al., 2008; and Harkness, Donald R.,1968), respectively, for standard substrate 4-nitrophenyl phosphate. Although we can observe the **Phos-CN(S)** that co-aggregate with the enzymatic product due to aggregation-enhanced responsiveness, the molecularly dissolved portion of **Phos-CN(S)** is invisible (Figure 3b), indicating that the local concentration of the dispersive substrate in cell is lower than LOD (5 μM). Thus, to simplify the math, we assumed the intracellular concentration of substrate [S]<<K_M_ in this study. So, equation [4] is suitable to determine the rate of product formation in this study. Since the cellular uptake and the enzymatic conversion of substrate is ongoing simultaneously, we also assumed that the [S] in cells is in a dynamic equilibrium state. So, [S] and enzyme concentration [E] in cells are homeostatic. Thus, we have:

[5] $$\int_{0}^{t}\frac{d[P]}{dt}=[P]=\frac{\text{kcat}}{Km}[E][S]t$$

Here t is the incubation time with substrate. So:

[6] $$\frac{\left[P\right]}{\left[S\right]}=\frac{\text{kcat}}{Km}[E]t$$

Thus, with the same incubation time, the ratio of [P]/[S] between different cell populations can be written as:

[7] $${\left(\frac{\left[P\right]}{\left[S\right]}\right)}_{1}:{\left(\frac{\left[P\right]}{\left[S\right]}\right)}_{2}={\left(\frac{\text{kcat}}{Km}\right)}_{1}{\left[E\right]}_{1}:{\left(\frac{\text{kcat}}{Km}\right)}_{2}{\left[E\right]}_{2}$$

If the enzyme concentration [E] between different cell population is nearly identical ([E]_1_=[E]_2_):

[8] $${\left(\frac{\left[P\right]}{\left[S\right]}\right)}_{1}:{\left(\frac{\left[P\right]}{\left[S\right]}\right)}_{2}={\left(\frac{\text{kcat}}{Km}\right)}_{1}:{\left(\frac{\text{kcat}}{Km}\right)}_{2}$$

Thus, the relative enzyme catalytic efficiency (kcat/K_M_) of phosphatase in the cells from different cell populations can be approximately quantified through comparing the product-to-substrate ratio ([P]/[S]).

### Image processing:

All images were opened and processed by ImageJ. Colocalization analysis was done by ImageJ using the colocalization threshold and coloc 2 plugin.

## Data Availability

All the data related to the work is available upon reasonable request to the corresponding author.
